# Gonorrheal Rheumatism1Read before the Surgical Section of the Buffalo Academy of Medicine, June 5, 1900.

**Published:** 1900-09

**Authors:** J. Henry Dowd

**Affiliations:** Buffalo, N. Y., 288 Franklin Street


### GONORRHEAL RHEUMATISM.[1]

BY J. HENRY DOWD, M. D., Buffalo, N. Y.

I USE the term gonorrheal rheumatism for no better name has as yet been found to characterise this complication of specific urethritis. It is an affection involving not only the joints but the theca of tendons, muscles and nerves, also the synovial bursæ, although the three latter varieties are rare. There seems to be no doubt that this affection is due to the absorption of gonococci, or their toxins from an abraded urethra. It is now conceded that the former are always present, this being demonstrated by cultures. Absorption from the vulva, vagina, cervix and endometrium has also been proven, but few cases are reported. The toughness of the epithelium of these parts, together with the fact that these structures are the ones generally involved in gonorrhea of the female, goes a long way in explaining why the latter class rarely have this complication. It is only in the subacute stage that we find this complication arising. This is accounted for by the swelling of the mucous membrane during the acute stage and also that it is the continued severe inflammatory action in the canal that causes the epithelium to become abraded, thus facilitating absorption.

In the five cases occurring in my own practice the earliest sign of involvement was seventeen days after the onset of the acute urethritis. The too early use of instruments is also an important factor in caus-

1. Read before the Surgical Section of the Buffalo Academy of Medicine, June 5, 1900.

ing solution of continuity of the epithelium and even the mucous membrane. I am almost positive that in my last case, thecitis was due to the use of sounds, the physician mistaking the trouble in the canal.

A certain class of individuals seems more prone to this complication than others. Thus we find that a father, mother or grandparents had gout, rheumatism, and the like. Of 376 cases tabulated the joints involved were as follows: knee, 136 times; tibiotarsal, 59 times; wrist, 43 times; fingers, 35 times; elbow, 25 times. In my own cases: knee, 1; elbow, 1; wrist, 1; metatarsus, 1; thecitis, 1. It is thus seen that the knee is the favorite joint. Pain and swelling of this joint during a subacute gonorrhea must be looked at with suspicion.

SYMPTOMS.

It will be unnecessary for me to go deeply into detail regarding the symptomatology of this affection, but rather a few brief and emphasised points should place this complication so in your mind that a mistake in its diagnosis would be practically impossible.

The joint involvement may be divided into two classes, viz.: monoarticular and polyarticular. The first, which is most frequent is ushered in by headache, general pains in the back, legs and several joints. These soon disappear, excepting in one joint, usually the knee, which becomes swollen, red and very tender, assuming a semiflexed position. If the limb is put at rest at once, so as to avoid irritation, the acute symptoms subside very shortly, but the exudation and swelling remain.

In favorable cases where rest and careful attention is given to the urethra, absorption of the exudate may take place in the course of six or seven weeks and a healthy joint remain. As long as there is any infiltration, forcible movement of the joint, as also irritation of the urethra will tend toward continuing the trouble or may cause an acute exacerbation. Although this will also disappear repeated occurrences of the same tend toward chronicity, which is liable to pass into hydrarthrosis.

Should suppuration occur this will manifest itself, by chills, increased fever, swelling, with pronounced tenderness and other symptoms indicating pus. At times the surrounding tissues become inflamed, edematous and tender and the capsule ruptures, the pus burrowing between the muscles and tendons. If recovery occurs it is accompanied by ankylosis, but this is the condition that is very liable to result in pyemia and death.

*Polyarticular.*—This may be easily mistaken for acute articular rheumatism, still differential symptoms are present which should at once clear any doubt in one's mind. The number of joints involved are less than in rheumatism; fever is not as high and the severa joints are attached simultaneously instead of in rotation or by metastasis, as in the former. Lastly, where the former (rheumatism) will respond to antirheumatics, the latter will not be effected in the least. As with monoarticular, this class can also become chronic, making the patient an almost helpless invalid for life.

*Hydrarthrosis.*—This, as already stated, is the result, as a general thing, of frequent relapses of an acute condition which to all appearances may have apparently subsided. The knee is the only joint so far observed in which this complication has been noted. There is no fever, ill health or inflammatory manifestations of the skin covering the joint, which is much increased in circumference and somewhat painful. The fluid, which is a pale yellowish-green, may be absorbed very rapidly, or the reverse may be true, and the case continued for months, resulting in plastic changes with deformities. Pathologically, König's classification is a good one to go by. He describes this infection under the head of hydrops, serofibrinous, empyemic and phlegmonous.

It is in accompanying joint troubles that we generally find the eye involved, not as a conjunctivitis, but an inflammation of the membrane of Descemet. It is usually accompanied by pain and should the iris become involved there are symptoms referable to this condition. Involvement of the theca, muscles, tendons and nerves seem to be very rare, but from personal contact with a case of the former some time ago, I am led to think that this is a complication which might be present and easily mistaken for something else. At times it is accompanied by pain most excruciating, where at others this phenomena is not of much consequence. There is one peculiarity noticed, and the same may be said of myositis—namely, the femoral region is the part suffering from the attack. In my case but one group of muscles (flexors) seemed to be involved and when at rest there was no pain, but on attempting to move it was very marked. This localisation in one group of muscles is explainable by the fact of the close relation of the sheaths with possible communication, at or near their origin or insertion.

Lydston reports a case of involvement of both sciatica nerves accompanying a gonorrheal affection, this subsiding as resolution took place, only to return at a second infection. If deep pressure is made over the affected area, pain is usually much marked.

*Myositis*—Muscular involvement is also a rare complication which seems to have a special affinity for the femoral region; in fact, Professor Eichhorst, of Zurich, says "in every case so far observed it was the femoral region that was invaded." He also states that this condition is due to the gonococci and not the staphylococci. There is a distinct swelling of the part which is hard and slightly painful. Although the severer symptoms gradually disappear, a hardness remains for some time. Fever may or may not be present and the skin usually remains in a normal condition.

A guarded prognosis must be given in all joint troubles complicating gonorrhea. Good results may be looked for where appropriate treatment is commenced early,but no man can foretell what joint may become ankylosed or even destroyed, necessitating the loss of a limb.

### TREATMENT.

Before speaking of this in detail it may not be amiss to call attention to the facts that (1) we are dealing with an infection caused by the absorption of cocci or their toxins, the latter being in reach of destructive agents; (2) here, as in all other inflammations, our first and only rule must be rest to the part involved.

When a diagnosis is positively established the joint or joints must be rendered immobile at once. The smaller ones, where very little swelling can take place, undoubtedly the best mode of fixation will be plaster-of-Paris bandage. In the knee, for instance, it is advisable to fix by posterior splint and roller bandage,the latter being applied so that the joint is exposed where during the acute condition an ice bag must be kept constantly applied.

Immobility being assured attention should be directed to the urethra for the destruction of the offending agent.

This resolves itself into the cure of the inflammation with the least possible irritation. The time at which the complication develops plays the most important part in the mode of treatment that should be pursued. If seen early, *i.e.*, within three or four weeks of the acute urethral infection, by all odds medication by the patient himself is the ideal treatment. On the other hand, if four weeks or more has elapsed, local treatment by the attendant is the only hope of a successful termination of the urethral involvement. It will be impossible to dilate upon this subject further than to say, daily injections of silver nitrate should be used, commencing with a strength of 1-12,000 in distilled water. The solution must be increased daily until a strength of 1-1000 is reached, if necessary, and these used without aid of any instrument in the canal[1].

While the urethra is under treatment, the patient should be carefully watched as to temperature, general condition and state of joint or joints involved. It is rare to find the smaller joints markedly enlarged from effusion, but the knee is often the reverse, the pressure causing much pain. When bulging is marked, under strict antiseptic precautions, puncture with evacuation of the fluid and flushing of the cavity with sterilised water yields the most happy results. This should be carried out in every case where the symptoms are at all severe. Should pus be found, an antiseptic solution (never bichloride) as Thiersch's should be substituted. In case of suppuration continuing and rupture of the capsule with disorganisation of the joint threatened, opening of the same and drainage is absolutely necessary. Ankylosis being prone to follow this condition, it is advisable to have the limb placed in the best possible position, so that the patient may not be too greatly handicapped in after life. When destruction of the articulai surfaces has progressed to a degree where the joint is entirely destroyed, resection with resulting partial, if not complete, ankylosis must be accepted. Although hydrarthrosis is rarely followed by serious results, treatment is often prolonged before complete recovery ensues, and at times this is unattainable. If the case is seen early blisters by cantharis or iodine, pressure by the rubber bandage and rest gives the best results. Even these may fail to bring about absorption of the fluid and puncture may be necessary.

When this is practised the most careful antiseptic precautions are all important, and as soon as the joint is emptied the puncture opening should be sealed and pressure and rest enforced. This may fail, the joint refilling and soon attain the previous size. In this condition and after repeated trials have been given the afore mentioned, the joint may be emptied and after thorough washing, to remove all débris, a 5 per cent. solution of carbolic acid can be injected, which should be carefully distributed by manipulation of the fingers externally. The joint must again be fixed, pressure applied and rest insisted upon. Pain can be relieved by morphine or heroine, but great care should be used to prevent the patient from becoming a constant user of these drugs. The general condition of the patient often needs attention, but tonics are the only drugs from which any results may be hoped for.

1. Dowd: Buffalo Med. Jour., August, 1897; Medical Record, September, 1897; British Med. Jour., London, Eng., October, 1897; Review of Reviews, November, 1897; American Jour. Dermatology and Genito-Urinary Diseases, October, 1897.

Case I.—Gonorrhea, first attack. No instrumentation of the urethra. About three weeks after, noticed right knee swelling; very painful. Ordered to bed with splint and ice bag. During the next six weeks, the temperature ranged from 99½° to 101°, the joint continuing to swell, causing much suffering. On the tenth day after involvement, with the assistance of Dr. J. F. Myer, I opened the joint, removing over a pint of fluid. About the eighth week the pain having entirely disappeared, massage, with passive motions was commenced. At the end of about three months, motion perfectly normal, and the swelling and the thickening had entirely disappeared. About the fifth or sixth week, the parents of the young man, asked for consultation. A surgeon of the city was called who advised amputation.

Case II.—Consultation—A.G., aged 28; single; about four weeks ago contracted gonorrhea. One week ago noticed swelling of the left wrist, accompanied by severe pain and warmth of the body (fever). At my visit the following condition was found: wrist much enlarged, flexed and very painful to the touch or on attempting movement. Temperature, 100°F.; profuse uretheral discharge and urine passed into two glasses, showed marked opacity of both. Treatment had consisted of balsam copabia and an injection. The wrist was upon a pillow and flax seed poultices were being constantly applied. The joint was put upon a splint for a couple of days and a solution applied to, if possible, reduce some of the swelling before applying the plaster.

On April 22d, the joint was firmly fixed with plaster and treatment begun for the cure of the urethral trouble. Within five or six days thereafter the temperature became normal and the pain had almost entirely disappeared, except when the fingers were moved.

Daily irrigation of the urethra with silver solutions was carried out and on the seventeenth day the pain having entirely subsided the plaster was removed and a roller bandage applied. During the following two weeks passive motion, following massage, with oleate of mercury ointment was practised, at the end of which time there was normal motion, no swelling, no urethral discharge and both glasses of urine was perfectly clean. In this case I failed to ask if any instruments had been used during the acute condition, but the patient told me very strong injections were given him. The time occupied for complete resolution in the joint was about four weeks. There was no rheumatic history in this family.

Case III.—Applied for treatment one week after commencement of an acute attack of gonorrhea. A physician was consulted, on appearance of discharge, who said there was a chancre in the urethra, and passed a sound. This was repeated twice during the week. Prescribed appropriate treatment and the patient left for New York, returning in about a week. At this time the discharge had materially decreased and the urine was becoming clear. During the next week he indulged freely in beer and on his visit, presented the following conditions: severe pain in the back, extending down the femoral region of the left side as far as the knee; right knee one-half inch larger than the left, red and quite painful. The condition was explained to the patient and the knee painted with iodine and a tight bandage applied, with instructions to remain quiet and continue medicines. Four days later the knee had assumed a normal condition and the pain was absent, but the flexors of the left femoral region were painfully involved to such an extent that walking was almost impossible. This continued for about ten days, when the pain moved to the popliteal space, at the same time involving an area directly over the sartorius muscle. Rest was enforced as much as possible and in the course of a couple of weeks the pain had entirely disappeared, also the discharge, the urine becoming practically clean, except a few small shreds which persisted. In about a month the canal was examined and two strictures, number 20 F, were discovered about two inches back from the meatus. These were dilated. There is no doubt that the use of a sound was the cause of the rheumatic condition, and also the strictures were of traumatic origin.

